# Information Theoretic Measures and Their Applications

**DOI:** 10.3390/e22121382

**Published:** 2020-12-07

**Authors:** Osvaldo A. Rosso, Fernando Montani

**Affiliations:** 1 Instituto de Física, Universidade Federal de Alagoas, Maceió, Alagoas 57072-970, Brazil; 2 Instituto de Física La Plata, CONICET-Universidad Nacional de la Plata, La Plata, Diagonal 113 entre 63 y 64, La Plata 1900, Argentina

## Special Issue Information

The concept of entropy, an ever-growing physical magnitude that measured the degree of decay of order in a physical system, was introduced by Rudolf Clausius in 1865 through an elegant formulation of the second law of thermodynamics. Seven years later, in 1872, Ludwig Boltzmann proved the famous *H*-theorem, showing that the quantity *H* always decreases in time, and in the case of perfect gas in equilibrium, the quantity *H* was related to Clausius’ entropy S. The dynamical approach of Boltzmann, together with the elegant theory of statistical ensembles at equilibrium proposed by Josiah Willard Gibbs, led to the Boltzmann–Gibbs theory of statistical mechanics, which represents one of the most successful theoretical frameworks of physics. In fact, with the introduction of entropy, thermodynamics became a model of theoretical science.

In 1948, Claude E. Shannon developed a “statistical theory of communication”, taking ideas from both logic and statistics that in turn opened new paths for research. The powerful notion of information entropy played a major part in the development of new statistical techniques, overhauling the Bayesian approach to probability and statistics. It provided powerful new techniques and approaches on several fields of science, extending and shedding new light on the field.

In the space of a few decades, chaos theory has jumped from the scientific literature into the popular realm, being regarded as a new way of looking at complex systems like brains or ecosystems. It is believed that the theory manages to capture the disorganized order that pervades our world. Chaos theory is a facet of the complex systems paradigm having to do with determinism randomness. In 1959, Kolmogorov observed that Shannon’s probabilistic theory of information could be applied to symbolic encodings of the phase–space descriptions of physical nonlinear dynamical systems so that one might characterize a process in terms of its Kolmogorov–Sinai entropy. Pesin’s theorem in 1977 proved that, for certain deterministic nonlinear dynamical systems exhibiting chaotic behavior, an estimation of the Kolmogorov–Sinai entropy is given by the sum of the positive Lyapunov exponents for the process. Thus, a nonlinear dynamical system may be viewed as an information source from which information-related quantifiers may help to characterize and visualize relevant details of the chaotic process.

In general speaking terms, physics as well as other scientific disciplines, such as biology or finance, can be considered observational sciences, that is, they try to infer properties of an unfamiliar system from the analysis of a measured time record of its behavior (time series). Dynamical systems are systems that evolve in time. In practice, in general, one may only be able to measure a scalar time series X(t) which may be a function of variables $V=\{v_{1},v_{2},\cdots ,v_{k}\}$ describing the underlying dynamics (i.e., dV/dt=f(V)). Then, the natural question is, how much we can learn from X(t) about the dynamics of the system. In a more formal way, given a system, be it natural or man-made, and given an observable of such a system whose evolution can be tracked through time, a natural question arises: how much information is this observable encoding about the dynamics of the underlying system ?

The information content of a system is typically evaluated via a probability distribution function (PDF) P describing the apportionment of some measurable or observable quantity, generally a time series $X\left(t\right)=\left\{x_{t},t=1,\cdots ,M\right\}$. Quantifying the information content of a given observable is therefore largely tantamount to characterizing its probability distribution. This is often done with a wide family of measures called Information Theory quantifiers (i.e., Shannon entropy and generalized entropy forms, relative entropy, Fisher information, statistical complexity, etc.). Thus, information theory quantifiers are measures that are able to characterize the relevant properties of the PDF associated with these time series, and, in this way, we should judiciously extract information on the dynamical system under study.

The evaluation of the information theory quantifiers supposes some prior knowledge about the system; specifically, a probability distribution associated with the time series under analysis should be provided beforehand. The determination of the most adequate PDF is a fundamental problem because the PDF P and the sample space Ω are inextricably linked. Many methods have been proposed for a proper selection of the probability space (Ω,P).

Usual methodologies assign a symbol from a finite alphabet A to each time point of the series X(t), thus creating a *symbolic sequence* that can be regarded as a *non causal coarse grained* description of the time series under consideration. As a consequence, order relations and the time scales of the dynamics are lost. The usual histogram technique corresponds to this kind of assignment. *Time causal information* may be duly incorporated if information about the past dynamics of the system is included in the symbolic sequence, i.e., symbols of alphabet A are assigned to a portion of the phase-space or trajectory.

In particular, Bandt and Pompe (BP) [“Permutation Entropy: A Natural Complexity Measure for Time Series.” *Phys. Rev. Lett. 1972, 88, 174102*] introduced a simple and robust symbolic methodology that takes into account the time causality of the time series (causal coarse grained methodology) by comparing neighboring values in a time series. The symbolic data are *(i)* created by ranking the values of the series; and *(ii)* defined by reordering the embedded data in ascending order, which is tantamount to a phase space reconstruction with embedding dimension (pattern length) D≥2, D∈N and time lag τ∈N. In this way, it is possible to quantify the diversity of the ordering symbols (patterns) derived from a scalar time series. Note that the appropriate symbol sequence arises naturally from the time series, and no model-based assumptions are needed. In fact, the necessary “partitions” are devised by comparing the order of neighboring relative values rather than by apportioning amplitudes according to different levels. This technique, as opposed to most of those in current practice, takes into account the temporal structure of the time series generated by the physical process under study. As such, it allows us to uncover important details concerning the ordinal structure of the time series and can also yield information about temporal correlation. Furthermore, the ordinal patterns associated with the PDF are invariant with respect to nonlinear monotonous transformations. Accordingly, nonlinear drifts or scaling artificially introduced by a measurement device will not modify the estimation of quantifiers, a nice property if one deals with experimental data.

Among other methodologies of non causal coarse grained type, we can mention frequency counting, procedures based on amplitude statistics, binary symbolic dynamics, Fourier analysis, or wavelet transform. The suitability of each of the proposed methodologies depends on the peculiarity of data, such as stationarity, length of the series, the variation of the parameters, the level of noise contamination, etc. In all these cases, global aspects of the dynamics can be somehow captured, but the different approaches are not equivalent in their ability to discern all relevant physical details.

In relation to other quantifiers, we can mention those based on mutual information which rigorously quantifies, in units known as “bits”, how much information the value of one variable reveals about the value of another. This is a dimensionless quantity that can be thought of as the reduction in uncertainty about one random variable given knowledge of another. Fisher information, which predates the Shannon entropy, and the more recent statistical complexities have also proved to be useful and powerful tools in different scenarios, allowing in particular to analyze time series and data series independently of their sources. The Fisher information measure can be variously interpreted as a measure of the ability to estimate a parameter, as the amount of information that can be extracted from a set of measurements, and also as a measure of the state of disorder of a system or phenomenon.

Among the most recent entropy proposals, we can mention approximate entropy; sample entropy; delayed permutation entropy; and permutation min-entropy. That is, different methodologies have been used to understand the mechanisms behind information processing. Among those, there are also methods of frequency analysis like wavelet transform (WT), which distinguishes itself from others due to the high efficiency when dealing with feature extraction. The “wavelet analysis” is the appropriate mathematical tool to analyze signals in the time and frequency domain. All these measures have important applications not only in physics but also in quite distinct areas, such as biology, medicine, economy, cognitive sciences, numerical and computational sciences, big data analysis, complex networks, and neuroscience.

In summary, in the present Special Issue, manuscripts focused on any of the above-mentioned “Information Theoretic Measures as Mutual Information, Permutation Entropy Approaches, Sample Entropy, Wavelet Entropy and its Evaluations”, as well as its interdisciplinary applications, are more than welcome.

In this special issue, a series of articles under the common denominator of *Theoretical Information Measures and their applications* is presented. In particular, a brief description of the content of each of the papers included is given below.

## The Contributions

- *Attention to the Variation of Probabilistic Events: Information Processing with Message Importance Measure.* By She, R.; Liu, S.; Fan, P. [1]
  Different probabilities of events attract different attention in many scenarios such as anomaly detection and security systems. To characterize the events’ importance from a probabilistic perspective, the message importance measure (MIM) is proposed as a kind of semantics analysis tool. Similar to Shannon entropy, the MIM has its special function in information representation, in which the parameter of MIM plays a vital role. Actually, the parameter dominates the properties of MIM, based on which the MIM has three work regions where this measure can be used flexibly for different goals. When the parameter is positive but not large enough, the MIM not only provides a new viewpoint for information processing but also has some similarities with Shannon entropy in the information compression and transmission. In this regard, the present work first constructs a system model with message importance measure and proposes the message importance loss to enrich the information processing strategies. Moreover, the message importance loss capacity is proposed to measure the information importance harvest in a transmission. Furthermore, the message importance distortion function is discussed to give an upper bound of information compression based on the MIM. Additionally, the bit rate transmission constrained by the message importance loss is investigated to broaden the scope for Shannon information theory.
- *Melodies as Maximally Disordered Systems under Macroscopic Constraints with Musical Meaning.* By Useche, J.; Hurtado, R. [2]
  One of the most relevant features of musical pieces is the selection and utilization of musical elements by composers. For connecting the musical properties of a melodic line as a whole with those of its constituent elements, the authors propose a representation for musical intervals based on physical quantities and a statistical model based on the minimization of relative entropy. The representation contains information about the size, location in the register, and level of tonal consonance of musical intervals. The statistical model involves expected values of relevant physical quantities that can be adopted as macroscopic constraints with musical meaning. The authors studied the occurrences of musical intervals in 20 melodic lines from seven masterpieces of Western tonal music. They found that all melodic lines are strictly ordered in terms of the physical quantities of the representation and that the formalism is suitable for approximately reproducing the final selection of musical intervals made by the composers, as well as for describing musical features as the asymmetry in the use of ascending and descending intervals, transposition processes, and the mean dissonance of a melodic line.
- *Discriminatory Target Learning: Mining Significant Dependence Relationships from Labeled and Unlabeled Data.* By Duan, Z.; Wang, L.; Mammadov, M.; Lou, H.; Sun, M. [3]
  Machine learning techniques have shown superior predictive power, among which Bayesian network classifiers (BNCs) have remained of great interest due to its capacity to demonstrate complex dependence relationships. Most traditional BNCs tend to build only one model to fit training instances by analyzing independence between attributes using conditional mutual information. However, for different class labels, the conditional dependence relationships may be different rather than invariant when attributes take different values, which may result in classification bias. To address this issue, the authors propose a novel framework, called discriminatory target learning, which can be regarded as a trade-off between probabilistic models learned from unlabeled instances at the uncertain end and that learned from labeled training data at the certain end. The final model can discriminately represent the dependence relationships hidden in unlabeled instances with respect to different possible class labels. Taking k-dependence Bayesian classifier as an example, experimental comparison on 42 publicly available datasets indicated that the final model achieved competitive classification performance compared to state-of-the-art learners such as Random forest and averaged one-dependence estimators.
- *Structure Extension of Tree-Augmented Naive Bayes.* By Long, Y.; Wang, L.; Sun, M. [4]
  Due to the simplicity and competitive classification performance of the naive Bayes (NB), researchers have proposed many approaches to improve NB by weakening its attribute independence assumption. Through the theoretical analysis of Kullback–Leibler divergence, the difference between NB and its variations lies in different orders of conditional mutual information represented by these augmenting edges in the tree-shaped network structure. In the present work, the authors propose to relax the independence assumption by further generalizing tree-augmented naive Bayes (TAN) from 1-dependence Bayesian network classifiers (BNC) to arbitrary k-dependence. Sub-models of TAN that are built to respectively represent specific conditional dependence relationships may “best match” the conditional probability distribution over the training data. Extensive experimental results reveal that the proposed algorithm achieves bias-variance trade-off and substantially better generalization performance than state-of-the-art classifiers such as logistic regression.
- *Permutation Entropy and Statistical Complexity Analysis of Brazilian Agricultural Commodities.* By de Araujo, F.; Bejan, L.; Rosso, O. A.; Stosic, T. [5]
  Agricultural commodities are considered perhaps the most important commodities, as any abrupt increase in food prices has serious consequences on food security and welfare, especially in developing countries. In this work, the authors analyze predictability of Brazilian agricultural commodity prices during the period after 2007/2008 food crisis. They use information theory based method Complexity/Entropy causality plane (CECP) that was shown to be successful in the analysis of market efficiency and predictability. By estimating information quantifiers permutation entropy and statistical complexity, they associate to each commodity the position in CECP and compare their efficiency (lack of predictability) using the deviation from a random process. The coffee market shows the highest efficiency (lowest predictability) while the pork market shows the lowest efficiency (highest predictability). By analyzing temporal evolution of commodities in the complexity–entropy causality plane, the authors observe that during the analyzed period (after 2007/2008 crisis) the efficiency of cotton, rice, and cattle markets increases, the soybeans market shows the decrease in efficiency until 2012, followed by the lower predictability and the increase of efficiency, while most commodities (8 out of total 12) exhibit relatively stable efficiency, indicating increased market integration in a post-crisis period.
- *Information Theory for Non-Stationary Processes with Stationary Increments.* By Granero-Belinchón, C.; Roux, S.; Garnier, N. [6]
  In the present contribution, the authors describe how to analyze the wide class of non-stationary processes with stationary centered increments using Shannon information theory. To do so, they use a practical viewpoint and define ersatz quantities from time-averaged probability distributions. These ersatz versions of entropy, mutual information, and entropy rate can be estimated when only a single realization of the process is available. We abundantly illustrate our approach by analyzing Gaussian and non-Gaussian self-similar signals, as well as multi-fractal signals. Using Gaussian signals allows them to check that their approach is robust in the sense that all quantities behave as expected from analytical derivations. Using the stationarity (independence on the integration time) of the ersatz entropy rate, they show that this quantity is not only able to fine probe the self-similarity of the process, but also offers a new way to quantify the multi-fractality.
- *Higher-Order Cumulants Drive Neuronal Activity Patterns, Inducing UP-DOWN States in Neural Populations.* By Baravalle, R.; Montani, F. [7]
  A major challenge in neuroscience is to understand the role of the higher-order correlations structure of neuronal populations. The dichotomized Gaussian model (DG) generates spike trains by means of thresholding a multivariate Gaussian random variable. The DG inputs are Gaussian distributed, and thus have no interactions beyond the second order in their inputs; however, they can induce higher-order correlations in the outputs. The authors propose a combination of analytical and numerical techniques to estimate higher-order, above the second, cumulants of the firing probability distributions. Their findings show that a large amount of pairwise interactions in the inputs can induce the system into two possible regimes, one with low activity (“DOWN state”) and another one with high activity (“UP state”), and the appearance of these states is due to a combination between the third- and fourth-order cumulant. This could be part of a mechanism that would help the neural code to upgrade specific information about the stimuli, motivating them to examine the behavior of the critical fluctuations through the Binder cumulant close to the critical point. The authors show, using the Binder cumulant, that higher-order correlations in the outputs generate a critical neural system that portrays a second-order phase transition.
- *Direct and Indirect Effects—An Information Theoretic Perspective.* By Schamberg, G.; Chapman, W.; Xie, S.; Coleman, T. [8]
  Information theoretic (IT) approaches to quantifying causal influences have experienced some popularity in the literature, in both theoretical and applied (e.g., neuroscience and climate science) domains. While these causal measures are desirable in that they are model agnostic and can capture nonlinear interactions, they are fundamentally different from common statistical notions of causal influence in that they (1) compare distributions over the effect rather than values of the effect and (2) are defined with respect to random variables representing a cause rather than specific values of a cause. The authors present here IT measures of direct, indirect, and total causal effects. The proposed measures are unlike existing IT techniques in that they enable measuring causal effects that are defined with respect to specific values of a cause while still offering the flexibility and general applicability of IT techniques. They provide an identifiability result and demonstrate application of the proposed measures in estimating the causal effect of the El Niño–Southern Oscillation on temperature anomalies in the North American Pacific Northwest.
- *New Fast ApEn and SampEn Entropy Algorithms Implementation and Their Application to Supercomputer Power Consumption.* By Tom$\overset{ˇ}{c}$ala, J. [9] Approximate Entropy and especially Sample Entropy are frequently used algorithms recently for calculating the measure of complexity of a time series. A lesser known fact is that there are also accelerated modifications of these two algorithms, namely Fast Approximate Entropy and Fast Sample Entropy. All these algorithms are effectively implemented in the R software package TSEntropies. This paper contains not only an explanation of all these algorithms, but also the principle of their acceleration. Furthermore, the paper contains a description of the functions of this software package and their parameters, as well as simple examples of using this software package to calculate these measures of complexity of an artificial time series and the time series of a complex real-world system represented by the course of supercomputer infrastructure power consumption. These time series were also used to test the speed of this package and to compare its speed with another R package pracma. The results show that TS Entropies are up to 100 times faster than pracma and another important result is that the computational times of the new Fast Approximate Entropy and Fast Sample Entropy algorithms are up to 500 times lower than the computational times of their original versions. At the very end of this paper, the possible use of this software package TS Entropies is proposed.
- *Composite Multiscale Partial Cross-Sample Entropy Analysis for Quantifying Intrinsic Similarity of Two Time Series Affected by Common External Factors.* By Li, B.; Han, G.; Jiang, S.; Yu, Z. [10]
  In this paper, the authors propose a new cross-sample entropy, namely the composite multiscale partial cross-sample entropy (CMPCSE), for quantifying the intrinsic similarity of two time series affected by common external factors. First, in order to test the validity of CMPCSE, they apply it to three sets of artificial data. Experimental results show that CMPCSE can accurately measure the intrinsic cross-sample entropy of two simultaneously recorded time series by removing the effects from the third time series. Then, CMPCSE is employed to investigate the partial cross-sample entropy of Shanghai securities composite index (SSEC) and Shenzhen Stock Exchange Component Index (SZSE) by eliminating the effect of Hang Seng Index (HSI). Compared with the composite multiscale cross-sample entropy, the results obtained by CMPCSE show that SSEC and SZSE have stronger similarity. The authors believe that CMPCSE is an effective tool to study intrinsic similarity of two time series. View Full-Text Keywords: composite multiscale partial cross-sample entropy (CMPCSE); multiscale cross-sample entropy (MCSE); time series; stock indices.
- *Effects of Tau and Sampling Frequency on the Regularity Analysis of ECG and EEG Signals Using ApEn and SampEn Entropy Estimators.* By Espinosa, R.; Talero, J.; Weinstein, A. [11]
  Electrocardiography (ECG) and electroencephalography (EEG) signals provide clinical information relevant to determining a patient’s health status. The nonlinear analysis of ECG and EEG signals allows for discovering characteristics that could not be found with traditional methods based on amplitude and frequency. Approximate entropy (ApEn) and sampling entropy (SampEn) are nonlinear data analysis algorithms that measure the data’s regularity, and these are used to classify different electrophysiological signals as normal or pathological. Entropy calculation requires setting the parameters r (tolerance threshold), m (immersion dimension), and τ (time delay), with the last one being related to how the time series is downsampled. In this study, we showed the dependence of ApEn and SampEn on different values of τ, for ECG and EEG signals with different sampling frequencies ($F_{s}$), extracted from a digital repository. We considered four values of $F_{s}$ (128, 256, 384, and 512 Hz for the ECG signals, and 160, 320, 480, and 640 Hz for the EEG signals) and five values of τ (from 1 to 5). We performed parametric and nonparametric statistical tests to confirm that the groups of normal and pathological ECG and EEG signals were significantly different (*p* < 0.05) for each F and τ value. The separation between the entropy values of regular and irregular signals was variable, demonstrating the dependence of ApEn and SampEn with $F_{s}$ and τ. For ECG signals, the separation between the conditions was more robust when using SampEn, the lowest value of $F_{s}$, and τ larger than 1. For EEG signals, the separation between the conditions was more robust when using SampEn with large values of $F_{s}$ and τ larger than 1. Therefore, adjusting τ may be convenient for signals that were acquired with different $F_{s}$ to ensure a reliable clinical classification. Furthermore, it is useful to set τ to values larger than 1 to reduce the computational cost.
